# Is National Resident Matching Program Rank Predictive of Resident Performance or Post-graduation Achievement? 10 Years at One Emergency Medicine Residency

**DOI:** 10.5811/westjem.2019.4.40602

**Published:** 2019-06-13

**Authors:** Jessica Wall, Scott R. Votey, Thomas Solomon, David L. Schriger

**Affiliations:** * University of California, Los Angeles, Department of Emergency Medicine, Los Angeles, California; † University of Washington, Department of Emergency Medicine, Seattle, Washington

## Abstract

**Introduction:**

Each year residency programs expend considerable effort ranking applicants for the National Residency Matching Program (NRMP). We explored the relationship between residents’ NRMP rank list position as generated at our institution and their performance in residency and post-graduation to determine whether such efforts are justified.

**Methods:**

Faculty who were present for the 10 consecutive study years at an allopathic emergency medicine residency retrospectively evaluated residents on their overall performance, medical knowledge, and interpersonal skills. Residency graduates were surveyed regarding their current position, hours of clinical practice, academic, teaching and leadership roles, and publications. We compared match position to performance using graphical techniques as the primary form of analysis.

**Results:**

Ten faculty evaluated the 107 residents who graduated from the program during these 10 years by class year. Eighty-four residents responded to the survey. In general, we found little correlation between NRMP rank and faculty rank of resident performance. There was also little correlation between position in the NRMP rank list and the probability of having an academic career, publishing research, or having a teaching or leadership role.

**Conclusion:**

We found that the position on our NRMP rank list was of little value in predicting which residents would do best in residency or take on academic or leadership roles once graduated. Residencies should evaluate the processes they use to generate their rank list to determine whether the ranking process is sufficiently predictive to warrant the effort expended.

## INTRODUCTION

Each year residencies across the United States participate in a time-intensive application process with three principal purposes: 1) to educate graduating medical student applicants about the residency program in a way that is both positive and realistic; 2) to identify applicants who would be a poor fit with the program and should not be ranked in the match; and 3) to differentiate highly desirable applicants from less desirable ones. While the first two objectives are necessary and feasible, the third objective poses a challenge and, in our experience, is very time intensive. Existing evidence suggests that programs are not particularly successful at determining which of their best performing medical student applicants will continue to be top performers in residency and ordering their rank lists accordingly.^1^–^3^ However, to our knowledge no study has explored the ability of the National Residency Matching Program (NRMP) rank to predict post-residency performance.

Our goal was to determine whether the NRMP applicant ranking process correlated with resident clinical performance, which has been studied on a few occasions,^1^–^7^ and post-graduation outcomes. This study was based on the ranking process currently conducted at a dual site emergency medicine (EM) residency program. We believe that if programs are unable to show that the energy expended ranking residents in a detailed manner produces important benefits to the program, then the faculty hours freed up by a less time-consuming method of ranking applicants could be redirected toward activities that improve the resident experience.

## METHODS

The overall design of our study was to compare the NRMP rank list generated for each class over a 10-year period to residency and post-graduation performance of the residents. This was achieved via the comparison of the rank-list position by class with attending evaluation and post-graduation survey data.

### Residency Recruitment Process

This two-part retrospective survey study took place in a dual site, four-year, allopathic EM residency program. The residency has been in place since 1978 with the mission of producing future leaders of EM in clinical research, academia, and public health administration. Our program has the luxury of having many more applicants (≈750) than positions (≈11 at the time of the study), so an applicant’s position on the rank list greatly influences his or her likelihood of joining our program.

During the time of this study, our selection process began with a crude screening of applications using objective criteria such as medical school attended and United States Medical Licensing Exam test scores to reduce the pool to 300–400 applicants. The residency recruitment committee reviewed these dossiers to identify the roughly 100 applicants who were offered an on-site interview. Each interviewee spent one of 10 interview days at the program, participating in activities that included three, 30-minute interviews with faculty members. The interviews were used to assess characteristics difficult to ascertain from an application dossier including interpersonal and communication skills, ambition, commitment to a career in EM, and humanitarian beliefs. Interviewing faculty members completed a standardized scoring form that included a global assessment placing the applicant in a rank list quintile or “do not rank.”

Additional faculty, residents, or staff (i.e., residency coordinators) who met the applicants during the interview day also submitted written comments. Based on the dossier, the interview ratings, and other comments, the program directors (PD) placed applicants in a tentative order. After each interview day they interdigitated additional applicants into the growing rank list. After concluding the interviews, PDs, faculty, and residents involved with recruitment reconsidered all applicants and held two full-day meetings to determine the final rank list submitted to the NRMP.

**Population Health Research Capsule**

What do we already know about this issue?

Residency programs expend considerable faculty time developing their National Residency Matching Program (NRMP) rank list. The value of fine tuning this list is unknown.

What was the research question?

Does NRMP rank order predict performance during residency or as a practicing physician?

What was the major finding of the study?

NRMP rank order did not predict performance during residency or career path in a meaningful way.

How does this improve population health?

This may motivate residencies to streamline their match list creation process.

### Study Design

In part 1 of this study, we obtained performance data for 10 years of residents using an electronically administered survey to elicit the opinions of longstanding, full-time faculty members who were present for the entire residency experience of these classes. Each faculty member independently ranked all of the residents within a graduating class from highest to lowest on three distinct measures based on residency milestones – overall performance, medical knowledge, and interpersonal skills – generating three rankings per resident per graduating class. The residents within a class were presented in random order, as were the classes, using software that allowed the faculty to move icons containing each resident’s name and photograph into the desired order. Ties were not permitted.

In part 2 of this study, we electronically surveyed former residents in the 10 graduating classes regarding clinical, leadership, and academic outcomes post-residency (Appendix 1). This survey was designed to capture various pathways of career advancement in EM based on promotion guidelines at local academic and community institutions. This study was approved by the institutional review board (IRB) at our home institution. All participants read an IRB-approved description of the study; their participation was deemed evidence of consent.

### Selection of Participants

All categorical EM residents who graduated from the program during 10 consecutive years were included. To maintain confidentiality of our participants, the specific years are not reported, but the 10-year period was within the time frame 1998–2013. We excluded residents who participated in our combined EM/pediatrics or EM/internal medicine training programs.

Faculty were eligible to participate if they were present for all years when these residents were in training, were not part of the study team, and were not involved in preparing the final NRMP rank order list, meaning that they were not a PD or an associate PD intimately involved in producing the final list. Faculty who interviewed applicants, met them at social events, or attended the rank meetings were eligible to participate provided they did not meet exclusion criteria.

### Outcome Measures and Analysis

We defined our outcomes as follows:

1. Faculty rank: For each resident attribute (overall performance, medical knowledge, interpersonal skills), we created an overall rank order for each class using the mean of the faculty ranks. We broke ties using the mode and, if there were still ties, the median.
2. Assessment of self-reported, post-residency professional activities: Based on their responses to the survey, residency graduates were classified into the mutually exclusive categories “Major Academic,” “Minor Academic,” “Community Practice,” and “Out of Emergency Medicine” with those in “Community Practice” further divided into “Leader,” “Teacher,” “Leader and Teacher,” or “Clinical Practice Only” using rules described in Appendix 2. These categories have not been previously described but were intended to group graduates by their general type of involvement in EM and, as such, have face validity.

Our independent variable was NRMP rank. Residents were assigned ranks first to Nth based on their position in the residency’s NRMP rank list relative to the other matched residents in their class. Residents taken outside the match were analyzed separately.

Our analysis was descriptive with the intention of visually depicting the degree of correlation between the NRMP rank order list, the faculty raters’ impression of each resident, and the graduates’ self-reported professional activities. Detailed graphics are used for this purpose as a method for visually assessing correlation. We examined the inter-rater reliability of the faculty rankings graphically, examining the distribution of deviations of each ranking from the average by rater and also examining the total squared deviations of each rater. We performed data management, analytics, and graph creation using Stata 14.2 (Stata Corp., College Station, Texas).

## RESULTS

Of the 14 eligible faculty members, 10 agreed to participate. The 10 classes had 107 categorical EM residency graduates, 95 of whom entered the residency through the match and had an NRMP rank. Class size varied from 9–12 residents over the 10 years, primarily due to variations in the number of residents participating in our combined EM/pediatrics and EM/internal medicine programs. Eighty-four (79%) residency graduates completed the survey of post-residency activity, 77 of whom entered the program through the NRMP match. Residents entering the program outside the match did so when the size of the residency increased or to replace residents lost to attrition.

**Figure 1:**
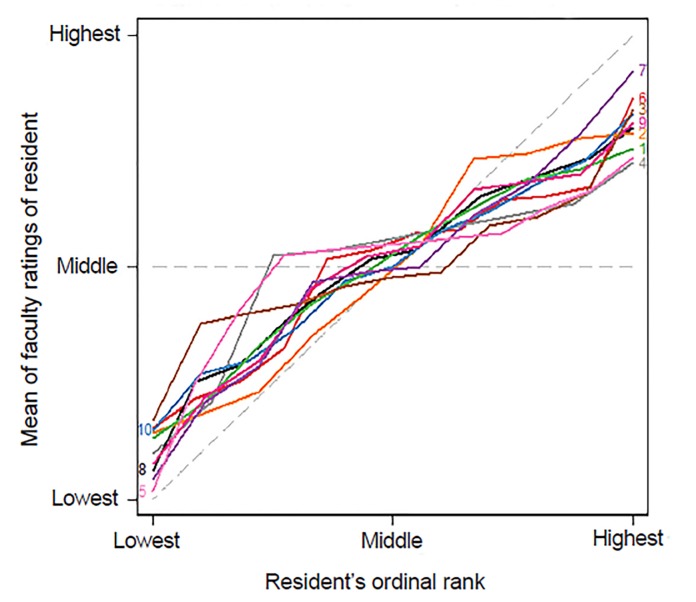
Faculty rating of residents’ mean rank vs ordinal rank by year. This graph illustrates inter-rater reliability for the faculty with regard to the overall performance question by class. The x-axis indicates the resident’s overall rank (See text for explanation). The y-axis is the mean of that resident’s faculty rankings. For each class (colored/numbered line), perfect agreement among faculty would result in a line that superimposes the 45° dashed line, as all faculty members would have ranked the overall highest ranked resident as 1, the next as 2, etc. Conversely, if there were perfect disagreement the lines would fall on the horizontal dashed gray line. Classes are labeled 1=earliest to 10=most recent.

**Figure 2:**
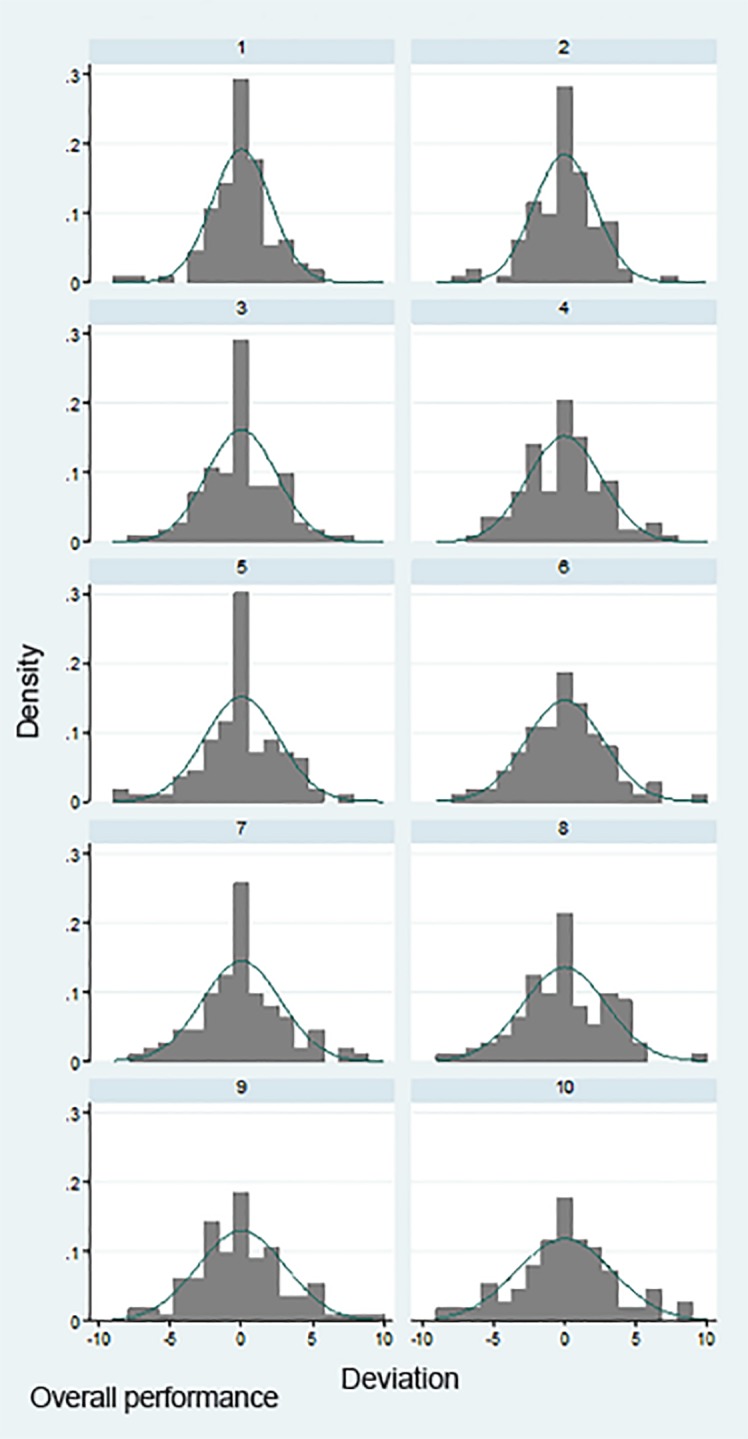
Deviation of faculty raters’ scores from average, by rater. Each histogram represents one faculty rater and shows the distribution of deviations between his or her rank and the mean rank of each of the 107 residents they ranked on overall performance. Raters are sorted from lowest (1) to highest (10) deviator, but there is no strong evidence that any raters were particularly better or worse than other raters. Histograms for the other questions are in the Appendix.

There was general agreement among faculty raters when ranking residents. Figure 1 provides support for the notion that faculty were generally consistent in the ratings of the residents. For most classes there was more agreement about who were the lowest- and highest-performing residents in a class, as the leftward and rightward sections of the curves are parallel to the 45^o^ (perfect agreement) line, and the middle section of the curve parallels the horizontal (no agreement) line. There was no evidence that raters were more consistent for more recent graduating classes (Figure 1 and Supplementary Figure 1). While there was variation in rankings among the faculty, no faculty member was an obvious outlier (Figure 2 and Supplementary Figure 2). Together these analyses suggest that the faculty were able to rank resident performance within each class with sufficient reliability to make these rankings meaningful.

**Figure 3:**
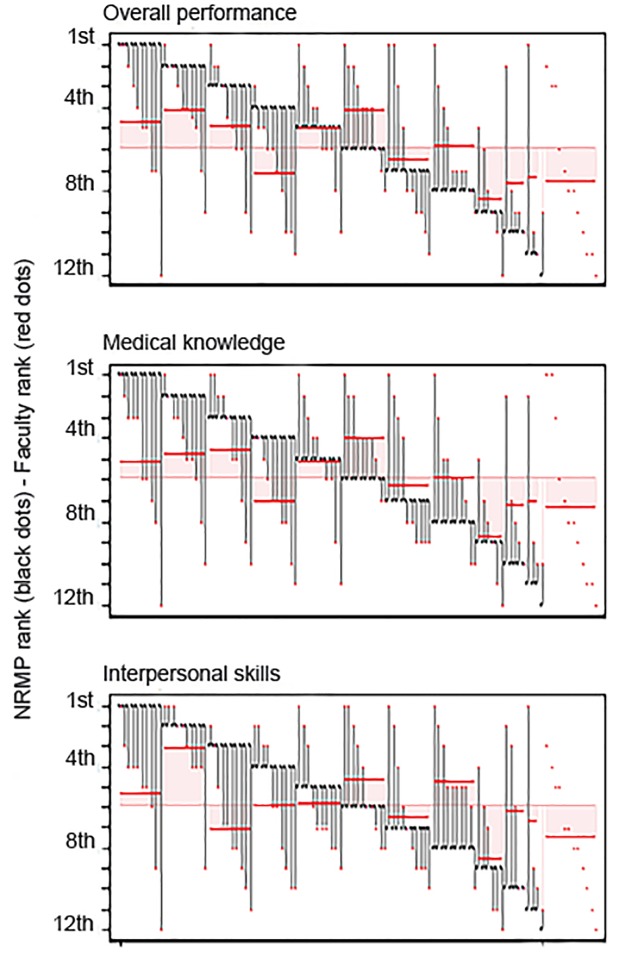
National Residency Matching Program (NRMP) rank and average faculty rank by resident, 1998–2013. This figure illustrates the NRMP rank order for each matched resident (black dot) and their average faculty rating (red dot) sorted by their NRMP rank. Faculty ratings for residents taken outside the match are shown on the far right. For each NRMP rank, the solid red bar segment represents the mean faculty rating of the residents who had that NRMP rank across all years, while the horizontal is the overal mean rank. The pink shading represents the divergence of the mean rating for the group of residents with that NRMP rank and the mean faculty mean rating for all residents.

While there is some evidence that residents taken higher in the NRMP match received higher performance ratings from the faculty, the association was quite weak. Figure 3 shows that top faculty-rated residents came from all parts of the rank order list and from residents taken outside the match. The same is true for residents with low faculty ratings. While there is some evidence that being in the top half of the NRMP match predicts having an above-average faculty rating for overall performance and medical knowledge (top two panels of Figure 3), there appears to be no correlation between NRMP rank order and faculty rating of interpersonal skills. This is confirmed by analysis of the distribution of deviations of NRMP rank and faculty rank (Supplementary Figure 3).

**Figure 4:**
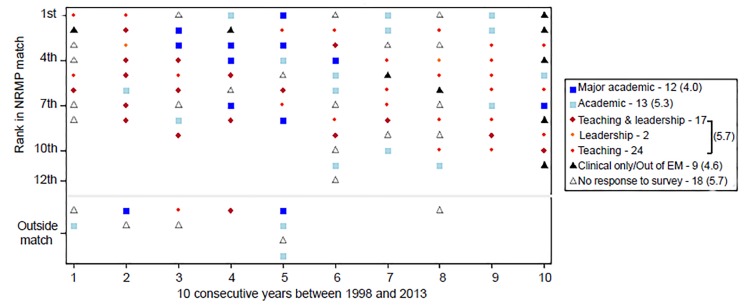
Professional outcome of graduates by year and National Resident Matching Program (NRMP) rank. Scatter plot of NRMP rank vs residency class based on post-graduation survey responses. Legend categories are defined in the text and Appendix 1. Following each category is the number in that category and the average rank in the NRMP match for that category. Parentheses in rightward box denote average class rank (of 12 positions).

We observed a similar pattern when we compared self-reported professional activity with NRMP rank order (Figure 4). Over the 10 years included in the study, the program produced 36% academics (30 of the 84 graduates who responded to the survey), 33% of whom came from the top three positions in the NRMP rank order for their residency class. Interestingly, another 17% of academics came from residents taken outside of the match; 71% (5 of 7) of residents taken outside of the match went into academics. While the average NRMP rank of “major academics” was highest (4.0), the next highest average NRMP rank (4.6) was for those graduates who no longer play an active role in academics, teaching, or leadership (n=8) or are no longer practicing EM (n=1). Those in clinical teaching and leadership roles tended to come from the middle NRMP ranks. Academics came from all parts of the rank order list, as did those who appear to have minimal continuing involvement in the specialty.

## DISCUSSION

Despite our institution’s rigorous applicant evaluation process, this study demonstrates that the relative position of a resident on our NRMP rank list was not meaningfully predictive of clinical performance during residency and participation in professional activities post-residency (Figures 3 and 4). While higher NRMP rank was somewhat predictive of a career in academics, this trend was not strong (Figure 4).

Our results reinforce the results of other investigations into the predictive value of the NRMP rank list. The majority of studies have shown that NRMP relative rank order correlates poorly with residency performance.^1^–^3^ Only one study found a positive correlation.^7^ Sklar and Tandberg (1996) demonstrated a positive correlation between NRMP relative rank order and faculty perception of 20 EM residents who graduated over a four-year period and found a positive correlation. Their results may differ from ours because they had smaller graduating classes (five per year) and ranked the four years of graduates together. Given the limited data and discrepant results, further investigation is warranted.

Many faculty hours are expended to determine the final rank order list. Given our findings, the time dedicated to a carefully ordered rank list appears to have low utility in predicting resident performance in residency and post-graduation, and less faculty-intensive methods should be considered.

## LIMITATIONS

Our study had notable limitations. We used a resident’s relative order from the rank list and relative class performance ranking by faculty to assess the predictive value of the rank order list. It is possible that other methods of analyzing NRMP rank would produce different results. Faculty evaluations of resident clinical performance were retrospective and thus subject to recall bias and recency bias. Our method for categorizing post-residency professional outcomes has not been previously described and only captures gross categories of activity. Prior studies have focused on in-residency performance and not categorized post-residency performance.

Fortunately, or unfortunately, there is no absolute definition or measure of “success.” Furthermore, there was an error with our post-graduate survey that had non-mutually exclusive choices (e.g., “0–5 publications” and “5–10 publications”), which would have been ambiguous for those with exactly five publications (Appendix 1). Finally, to protect anonymity, we did not take into account years since graduation with regard to publications; thus, residents from more recent graduating classes may not have the time to develop the criteria required to be considered a “major academic” or “leader.”

This study only investigated the performance and professional outcomes of the applicants who matched with us. Since we start with an applicant pool of approximately 750, interview roughly 100, and fill our 12 positions by an average of 50–60 on our rank list, we can only assess what the recruitment committee considered to be the top 8% of our applicants. We cannot address the utility of faculty time consumed determining who should be interviewed, nor the utility of time devoted to determining which applicants should be placed in the top vs the bottom half of our rank list, as we did not include those who were not matched at our program.

## CONCLUSION

In summary, there was a lack of strong correlation between our NRMP rank order and clinical performance during an EM residency, a finding similar to the majority of literature on the topic. Correlation between NRMP rank order and post-residency outcomes was also not strong. Given these findings, each residency will need to determine the appropriate amount of faculty labor used to formulate the NRMP rank order list while seeking ways to improve the predictive value of their rank lists and to identify and eliminate low-productivity faculty hours. We suspect that the process is insufficiently predictive to warrant current levels of expenditure of faculty time, which could be used more productively on other activities.

## Supplementary Information
