# Supplementary material for: Is National Resident Matching Program Rank Predictive of Resident Performance or Post-graduation Achievement? 10 Years at One Emergency Medicine Residency
Source: West J Emerg Med. 2019 Jun 13;20(4):641–6. doi: 10.5811/westjem.2019.4.40602 (PMC6625696; doi:10.5811/westjem.2019.4.40602)
Supplement: Supplementary file 1 [file wjem-20-641-s001.docx]

**Appendix**

**Appendix 1: Post-Graduate Survey**

1. Please enter the number of years since you graduated residency:____

2. Have you completed a fellowship or an additional residency (please select)? YES/NO

(If subject enters YES, then the following question is presented)

2b. Please enter the fellowship and/or additional residency program type(s):______________

3. For each of the following time intervals after EM residency, how many hours per week were spent as clinical time in an ED?

a. 0-5 years__________

b. 5-10 years__________

c. 10 years or greater___________

4. For each of the following time intervals after EM residency, how many hours per week were spent as clinical time a medical setting outside of an ED?

a. 0-5 years__________

b. 5-10 years__________

c. 10 years or greater___________

(If subject enters a number other than zero, then the following question is presented)

4b. Please enter the clinical work type(s) (type N/A if not applicable):______________

5. Please indicate your highest academic rank achieved (select one):

a. Staff Attending/Instructor

b. Assistant Professor

c. Associate Professor

d. Professor of Medicine

6. Please select the range reflecting your total number of academic publications (e.g. book chapters, peer-reviewed publications, non-peer reviewed professional publications)

a. 0-5__________

b. 5-10__________

c. Greater than 10___________

7. What do you consider to be your most important academic role (e.g. Research Director, Residency Director) (type N/A if not applicable)?_________________

8. Do you currently hold or have you held leadership roles (select all that apply):

1. In your practice group (e.g. ED Medical Director)?___
2. In your hospital (e.g. Chief of Medical Staff)?___
3. In an EM professional organizations (e.g. ACEP, AAEM, SAEM, etc)?___
4. In a state or federal organizations (e.g. FDA, CDC)?_________

9. Have you been actively involved in teaching EM to (please select all that apply)?

1. Prehospital care providers (e.g. EMTs, paramedics)___
2. Nurses___
3. Medical students or physicians in training (residents)___
4. Practicing physicians___

**Appendix 2:** **Coding rules for assessment of self-reported post-residency performance**

We begin by identifying graduate who are no longer involved in emergency medicine. Those with an academic title and greater than 10 publications or an academic title, fellowship and greater than 4 publications were classified as a “Major Academic”. Those with less then 10 publications and no fellowship or those with fellowship and 4 or less publications were classified as a “Minor Academic”. Non-academic titles were first assessed for having a academic role and greater than 4 publications, who were categorized as a “Minor Academic.” The remainder of those without an academic title were then classifed into as “Teachers” and “Leaders” by reporting both teaching or leadership activity respecitvely, or as “Leader and Teacher” by participating in both roles. Those practicing in the community with no teaching or leadership roles (8) were grouped with the 1 physician who was no longer involved with emergency medicine for analysis.


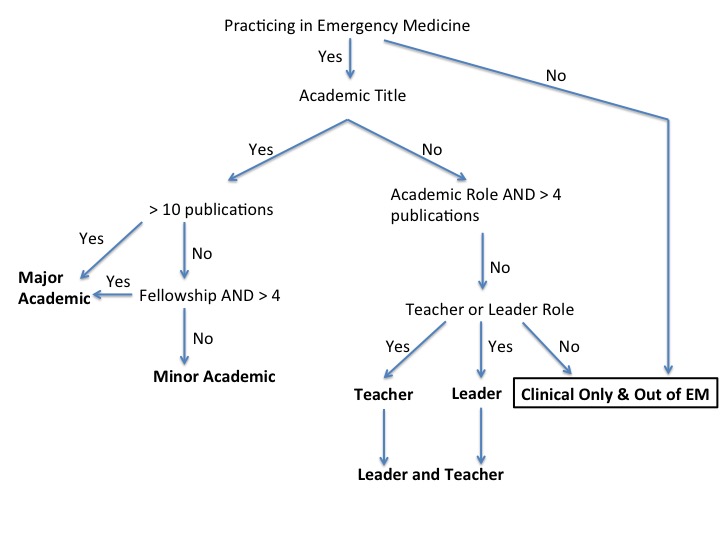


Yes

[Grab your reader’s attention with a great quote from the document or use this space to emphasize a key point. To place this text box anywhere on the page, just drag it.]
