# Supplementary material for: Is National Resident Matching Program Rank Predictive of Resident Performance or Post-graduation Achievement? 10 Years at One Emergency Medicine Residency
Source: West J Emerg Med. 2019 Jun 13;20(4):641–6. doi: 10.5811/westjem.2019.4.40602 (PMC6625696; doi:10.5811/westjem.2019.4.40602)
Supplement: Supplementary file 2 [file wjem-20-641-s002.docx]

Supplementary Figure 1: Each line represents a single rater. The points on the line are the sum of the squared deviations divided by the number of ratings for each graduating class. For years, such as the 9^th^, where faculty were in general agreement with each other, deviations are small and similar. In other years (e.g. 3^rd^, 8^th^, & 10^th^) there was more heterogeneity of opinion. There is no evidence that recall bias affected heterogeneity as there is little correlation of variance and year. There is also no evidence that one or more raters were consistenly spurious.

Supplementary Figure 2: Each histogram represents one faculty rater and shows the distribution of deviations between their rank and the mean rank of each of the 107 residents they ranked on medical knowledge (left panel) and interpersonal skill (right panel). Raters are presented in the same order as Figure 2 which is from the lowest to highest deviator on the overall performance question.

Supplementary Figure 3: This figure graphs the difference between NRMP and mean faculty rank for the 95 pairs of ratings for each question. If there were absolutely no correlation between the ranks the histograms would be normal (maroon lines). In the top panel (overall performance) the smaller deviations are overrepresented and larger deviations underrepresented compared to what would be expected if there were no correlation, but only by a small amount. Correlations are even weaker for the medical knowledge and interpersonal skills items.
